# Inducing Proactive Control Shifts in the AX-CPT

**DOI:** 10.3389/fpsyg.2016.01822

**Published:** 2016-11-22

**Authors:** Corentin Gonthier, Brooke N. Macnamara, Michael Chow, Andrew R. A. Conway, Todd S. Braver

**Affiliations:** ^1^Department of Psychology, LPNC UMR CNRS 5105, University of SavoyChambéry, France; ^2^Department of Psychology, CRPCC EA 1285, University of RennesRennes, France; ^3^Department of Psychology, Princeton University, PrincetonNJ, USA; ^4^Department of Psychological Sciences, Case Western Reserve University, ClevelandOH, USA; ^5^Division of Behavioral and Organizational Sciences, Claremont Graduate University, ClaremontCA, USA; ^6^Department of Psychology and Brain Sciences, Washington University in St. Louis, St. LouisMO, USA

**Keywords:** cognitive control, Dual Mechanisms of Control, proactive control, AX-CPT, strategy training, no-go manipulation

## Abstract

The Dual Mechanisms of Control (DMC) account (Braver, 2012) proposes two distinct mechanisms of cognitive control, proactive and reactive. This account has been supported by a large number of studies using the AX-CPT paradigm that have demonstrated not only between-group differences, but also within-subjects variability in the use of the two control mechanisms. Yet there has been little investigation of task manipulations that can experimentally modulate the use of proactive control in healthy young adults; such manipulations could be useful to better understand the workings of cognitive control mechanisms. In the current study, a series of three experiments demonstrate how individuals can be systematically biased toward and away from the utilization of proactive control, via strategy training and no-go manipulations, respectively. These results provide increased support for the DMC framework, and provide a new basis from which to examine group-based differences and neural mechanisms underlying the two control modes.

## Introduction

Humans rely heavily on cognitive control, the ability to use contextual information such as task goals to regulate behavior, particularly when adapting to the demands of complex tasks. However, understanding how cognitive control is implemented in practice has proven to be a major challenge in cognitive psychology. A growing body of research suggests that cognitive control is not, in fact, a unitary ability (e.g., Miyake et al., 2000; Engle and Kane, 2004; Badre, 2008; Banich, 2009). Instead, a recently developed framework – the Dual Mechanisms of Control (DMC) framework – suggests that cognitive control may operate via two distinct mechanisms: proactive control, referring to the active maintenance of contextual information to effectively bias cognitive processing in advance; and reactive control, referring to the selective retrieval of contextual information only when needed (Braver et al., 2007; Braver, 2012).

These two modes of control have different advantages and limitations. For example, proactive control is often more effective, but it is more demanding; it also typically requires predictive contextual cues to prepare the behavioral response in advance and depends on the validity of these cues (Braver et al., 2007). Proactive and reactive control are also associated with different patterns of neural activity: participants using proactive control demonstrate more anticipatory and sustained activity in the lateral prefrontal cortex (PFC), presumably reflecting the active maintenance of contextual information; conversely, reactive control is associated with increased transient activation throughout a wider frontoparietal network (e.g., Paxton et al., 2008; Braver et al., 2009).

Considerable support for the DMC framework has come from the study of variability in the use of cognitive control mechanisms, and especially variability across populations. For example, the tendency to use proactive control is reduced in young children (Chatham et al., 2009; Brahmbhatt et al., 2010), healthy older adults (Braver et al., 2001; Paxton et al., 2008; Bugg, 2014), older adults with Alzheimer-type dementia (Braver et al., 2005), and schizophrenic patients (Barch et al., 2001, 2003; MacDonald and Carter, 2003; Edwards et al., 2010). Conversely, it has been argued that individuals with high working memory capacity show an increased tendency toward proactive control (Redick, 2014; Richmond et al., 2015). Participants also demonstrate intra-individual variability related to affective-motivational factors, such as in the presence of incentives (Locke and Braver, 2008; Chiew and Braver, 2013) or following induction of positive affect (Dreisbach, 2006; van Wouwe et al., 2011).

The study of variability in the use of proactive and reactive control has provided support for the DMC framework in numerous ways (see Braver, 2012). However, it also has shortcomings: for example, comparing participants from different populations (such as schizophrenia patients and control participants) creates the potential to confound cognitive control with other factors that vary across the samples. Likewise, the use of affective-motivational manipulations, though critically important for the study of the interplay between emotion and cognition, complicates interpretations because affect and cognitive control demonstrate complex interactions (Botvinick and Braver, 2015; for an example, Braem et al., 2013). Thus, there is an important need to demonstrate that participants can be induced to shift control mechanisms through experimental manipulations that influence cognitive processing, such as strategic instructional emphasis or stimulus-induced attentional biases.

The introduction of experimental manipulations that can modulate control levels in healthy individuals would find wide application in a range of research questions. In the context of neuroimaging, for example, inducing participants to use one specific mode of control is required to properly isolate the neural substrates of proactive and reactive control. Being able to experimentally modulate cognitive control could also be very useful for studies interested in between-group comparisons: for example, healthy participants and patients with schizophrenia are known to differ in their tendency to spontaneously implement proactive control (Barch et al., 2001, 2003; MacDonald and Carter, 2003; Edwards et al., 2010), but it would be interesting to know if these two groups still demonstrate behavioral and neural differences when both induced to refrain from engaging in proactive control. Likewise, experimental manipulations would allow studies focused on individual differences in cognitive control to use the powerful experimental-correlational approach. For example, the higher performance of participants with high working memory capacity has been attributed to a higher tendency to implement proactive control (Redick, 2014; Richmond et al., 2015); this hypothesis could be confirmed by examining the relationship between working memory and performance when all participants are induced to use proactive control. With these applications in mind, the purpose of the present set of experiments was to validate two different experimental manipulations designed to induce shifts in the use of proactive control in healthy young adults, for use in further studies. These two manipulations were developed in the context of the AX-CPT, the paradigmatic task for the DMC framework.

### The AX-CPT Paradigm

The AX-CPT experimental paradigm has been used in the majority of past research investigating proactive and reactive control, partly because of its simple design and applicability in a wide range of populations (Barch et al., 2009; Chatham et al., 2009; Braver, 2012). The task is an adaptation of the classic Continuous Performance Test (CPT; Rosvold et al., 1956) developed to emphasize cognitive control by increasing control demands through the use of contextual cues (Servan-Schreiber et al., 1996; MacDonald, 2008). The AX-CPT requires participants to respond to a probe on the basis of a preceding cue. Each trial presents a cue letter followed by a probe letter presented after a delay period. Participants are tasked with making a target response when they detect the “AX” sequence (an A cue followed by an X probe), and a non-target response to all other letter sequences (AY trials: an A cue followed by any probe other than X, BX trials: any cue other than A followed by an X probe, and BY trials: any cue other than A followed by any probe other than X). A critical feature of the design is that A cues and X probes are strongly associated due to a large proportion of AX trials, leading both to an increased target expectancy following an A cue, and to a prepotent target response tendency when presented with an X probe.

The AX-CPT is highly sensitive to the control mode adopted by participants, in that proactive and reactive control are associated with different patterns of performance in the task (Braver et al., 2007). Participants using proactive control actively maintain contextual information related to the identity of the cue during the delay period, which allows them to prepare a target response if the cue is an A or a non-target response if the cue is not an A. This advance preparation strategy translates into better performance on BX trials, where the X is unlikely to serve as a convincing lure because the non-target response is already prepared, but worse performance on AY trials where participants incorrectly prepare a target response. Conversely, participants using reactive control selectively retrieve contextual information when the probe appears, which translates into heightened interference on BX trials, but better performance on AY trials.

### Inducing Cognitive Control Shifts in the AX-CPT

Several prior studies have successfully induced participants to preferentially engage proactive control in the AX-CPT by providing explicit training with a proactive strategy (Paxton et al., 2006; Braver et al., 2009; Edwards et al., 2010). The training involved specific labeling and utilization of contextual information from the cue, in order to anticipate and prepare the probe response ahead of time. Participants in the strategy training condition demonstrated a shift toward proactive control, indicated by improved performance on BX trials and decreased performance on AY trials (Paxton et al., 2006; Braver et al., 2009; Edwards et al., 2010), and by an increase in sustained activation in the dorsolateral PFC during the delay period of the task (Braver et al., 2009; Edwards et al., 2010). However, this method has only been tested in small samples from populations that are known to have cognitive control and AX-CPT impairments, such as older adults (Paxton et al., 2006; Braver et al., 2009) and schizophrenic patients (Edwards et al., 2010); strategy training could conceivably be inappropriate for participants who demonstrate a higher baseline level of proactive control. Therefore, our first objective was to evaluate the validity of proactive strategy training in a large non-clinical sample of young adults.

Fewer studies have tried to induce participants to reduce utilization of proactive control. Some studies have tried to present irrelevant distractor letters during the delay period of the AX-CPT to disrupt the active maintenance of contextual information and impair proactive control in participants (Braver et al., 2001; Dreisbach, 2006; Fröber and Dreisbach, 2016). However, it is unclear whether this manipulation actually induces participants to refrain from using proactive control, or whether it merely affects performance by decreasing the effectiveness of proactive control. Another study included no-go trials in the AX-CPT, in conjunction with a penalty manipulation (Braver et al., 2009). Participants were required to withhold their response on no-go trials (which occurred with low frequency and unpredictably during probe presentation), and were assessed a monetary penalty when they failed to do so. The hypothesis was that this manipulation would deter participants from using a proactive strategy, as preparing a response in advance would elicit more errors on the no-go trials. The no-go/penalty manipulation seemed successful in discouraging a proactive strategy, in that participants performed worse on BX trials in this condition and demonstrated a reduction in sustained activity in the dorsolateral PFC during the delay period of the task. This work has important shortcomings, however. First, it was only tested in a small sample of 16 participants in the context of a neuroimaging study. Second, the no-go manipulation failed to demonstrate significant effects on most behavioral indices of performance, presumably due to the small sample size. Third, it is unclear whether monetary penalties are a necessary component of the induction; it would be useful to determine whether no-go trials are effective in the absence of incentives, especially because this would allow for interpretations that do not require invocation of motivational mechanisms. Therefore, our second objective was to assess the validity of the no-go manipulation to bias participants away from proactive control in a larger sample of healthy young adults, without the use of monetary incentives.

In summary, the set of experiments presented here aimed to validate two methods for experimentally manipulating cognitive control in the AX-CPT. Experiment 1 tested whether strategy training can be used to increase the use of proactive control, Experiment 2 tested whether no-go trials can be used to decrease the use of proactive control, and Experiment 3 tested the combination of both manipulations in the same task. A significant feature of the experiments reported here is that they were performed at different universities, and that minor aspects of the AX-CPT (such as the presence of feedback after each trial, the precise length of the inter-stimulus interval, and the total number of trials) were varied across experiments: given that many variants of the AX-CPT have been used in past research, it was of interest to verify that our findings were not artifacts due to specific task or sample features. Procedural changes across experiments are documented in Appendix A. Relevant material for all experiments in this article (including task scripts and data files) can be accessed via the Open Science Framework platform at osf.io/6sqvw/.

## Experiment 1

The aim of Experiment 1 was to validate a version of the AX-CPT that induced proactive control by extending the strategy training method developed in earlier studies to a population of non-clinical young adults. Healthy young participants were expected to demonstrate a high level of proactive control at baseline (Paxton et al., 2008; Braver et al., 2009; Edwards et al., 2010), and strategy training was expected to bolster proactive control even further.

### Method

#### Statistical Power

The required sample size was estimated with a power analysis using G^∗^Power 3.1 (Faul et al., 2007). Based on prior work (Paxton et al., 2006; Edwards et al., 2010), we expected effect sizes ranging between η_p_^2^ = 0.15 and η_p_^2^ = 0.20 for the effect of strategy training on measures of interest. The necessary sample size to detect an effect size of η_p_^2^ = 0.15 with 0.95 power was estimated to be *N* = 77.

#### Participants

A sample of 78 students at Princeton University completed the experiment in exchange for partial course credit or payment ($12.00 per hour). All participants were native English speakers between the ages of 18 and 26 (*M* = 19.6 years, 30 male and 49 female). The experiment was approved by an ethics committee (Princeton University institutional review board); all participants provided written informed consent in accordance with the declaration of Helsinki.

#### Materials

##### AX-CPT

A version of the AX-CPT was constructed and implemented using E-prime software (Schneider et al., 2002). The version of the AX-CPT used in this study was based on the version used by Braver et al. (2009). Each trial began with a cue. The cue was a letter (any letter except X, K, or Y) presented in the center of the screen for 1000 ms. An unfilled inter-stimulus interval of 4000 ms followed. After the inter-stimulus interval, the probe appeared. The probe was a letter (any letter except A, K, or Y) presented in the center of the screen for 500 ms. Following the probe, a row of asterisks appeared during the 1000 ms inter-trial interval. Participants were instructed to press the target button with the middle finger of their right hand as quickly as possible whenever they observed an A cue followed by an X probe, and to press the non-target key with the index finger of the right hand as quickly as possible whenever they observed any other letter pair. Participants were instructed to respond only once they observed the second letter in the pair (i.e., the probe). Responses to the probe stimuli were recorded with a time limit of 1500 ms.

The proportions of trial types were based on those used by Richmond et al. (2015): 40% of the trials in each task block consisted of an A followed by an X (AX trials), 10% of the trials in each block consisted of an A followed by a letter other than X (pseudo-randomly selected; AY trials), 10% of the trials in each block consisted of a letter other than A (pseudo-randomly selected) followed by an X (BX trials), and 40% of the trials in each block consisted of a letter other than A (pseudo-randomly selected) followed by a letter other than X (pseudo-randomly selected; BY trials)^1^. Trials within each block were presented randomly.

##### Data processing

Error rates and average response times (RTs; computed for correct responses only) were recorded separately for each of the four trial types (AX, AY, BX, and BY). Three additional indices reflecting the use of proactive control were also computed: the *d′*-context, the A-cue bias, and the Proactive Behavioral Index (PBI). The first two indices, *d′*-context and A-cue bias, are based on signal detection theory (Stanislaw and Todorov, 1999). The *d′*-context index was calculated by computing a *d′* index from hits on AX trials and false alarms on BX trials as Z(H) - Z(F), with H representing hits on AX trials, F representing false alarms on BX trials, and Z representing the z-transform of a value. This measure reflects the ability of the participants to use contextual information from the cue to drive their answer on the probe (e.g., Barch et al., 2001). An A-cue bias measure was also calculated (Richmond et al., 2015) by computing a *c* criterion from hits on AX trials and false alarms on AY trials as 1/2*(Z[H] + Z[F]), with H representing hits on AX trials and F representing false alarms on AY trials^2^. This measure reflects the tendency of participants to make a target response following an A cue, independently of the identity of the probe. The third index was the PBI (Braver et al., 2009), calculated as (AY - BX)/(AY + BX). This index reflects the relative balance of interference between AY and BX trials: a positive PBI reflects higher interference on AY trials, indicating proactive control, whereas a negative PBI reflects higher interference on BX trials, indicating reactive control. The PBI was computed separately for error rates (based on average error rates on AY and BX trials) and for RTs (based on average RTs on AY and BX trials); a composite PBI was also computed by averaging the PBIs obtained for error rates and RTs after standardization. The standardization was performed by using the average and standard deviation calculated over both conditions of the AX-CPT to allow for comparison between the two. In order to correct for trials where error rates were equal to zero, a log-linear correction was applied to all error rate data prior to computing the *d′*-context, the A-cue bias and the PBIs (as in Braver et al., 2009; see also Hautus, 1995). This correction was applied as error rate = (number of errors + 0.5)/(number of trials + 1).

These three derived indices were of interest for two major reasons. First, they have been used in a large share of research based on the AX-CPT (e.g., Cohen et al., 1999; Barch et al., 2001; Braver et al., 2001, 2005, 2009; MacDonald and Carter, 2003; Paxton et al., 2008; Edwards et al., 2010; Richmond et al., 2015). Second, they provide an efficient way of summarizing the involvement of proactive control because they combine information from multiple trial types in a single measure. For example, one condition may be associated with slowing on both AY and BX trials, but BX trials may be slowed to a greater extent than AY trials; this shift in the balance between trial types is typically interpreted as a reduced use of proactive control (e.g., Barch et al., 2003; MacDonald and Carter, 2003; Braver et al., 2005; Redick and Engle, 2011), and directly translates into a lower PBI.

##### Strategy training

The strategy training procedure was based on the methods used by Paxton et al. (2006). The training comprised three steps: participants were informed that an X probe was very likely to follow an A cue; they were asked to mentally prepare for a target response during the inter-stimulus interval whenever they saw an A cue, and to prepare for a non-target response otherwise; and they were trained to implement this advance preparation strategy in a series of 60 practice trials. Details of the procedure and corresponding instructions are described in Appendix A.

#### Procedure

Participants underwent two experimental sessions 4–14 days apart in groups of up to six participants. During the first session, participants received detailed instructions for the AX-CPT. After receiving instructions, they viewed a demonstration of multiple trial types in which the correct response was displayed on the screen. Following the demonstration, participants completed 10 practice trials. The experimenter observed participants during the practice trials to ensure that they were completing the task correctly. All participants were able to understand the instructions and perform the task correctly during the practice. The participants then completed four blocks of 50 trials of the AX-CPT for a total of 200 trials (80 AX, 20 AY, 20 BX, 80 BY). The data from this first session were used as a baseline. During the second session, participants completed a first block of 50 trials identical to the first session, then received the strategy training and completed the corresponding practice trials. They then completed three blocks of 50 trials of the AX-CPT for a total of 150 trials; these trials were identical to the baseline version of the AX-CPT completed during the first session. The order of the two testing sessions could not be counterbalanced because a strategy training performed first would presumably have transferred to a baseline session performed second.

### Results

**FIGURE 1:**
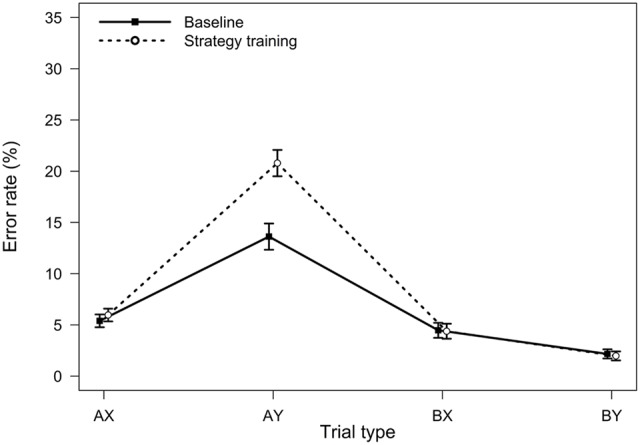
**Average error rates in the AX-CPT as a function of trial type and task condition (Experiment 1).** Error bars represent within-subjects standard errors of the mean (Morey, 2008).

**FIGURE 2:**
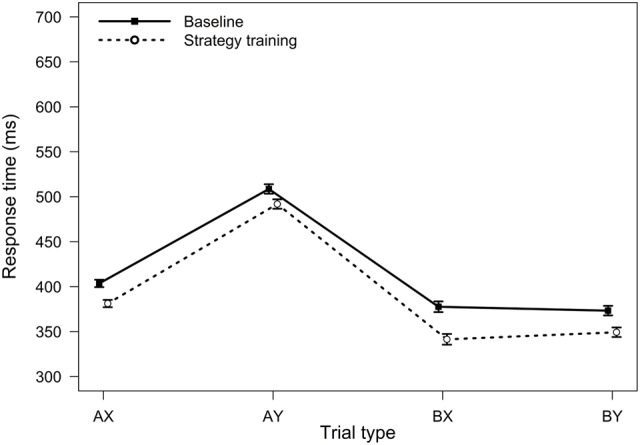
**Average response times in the AX-CPT as a function of trial type and task condition (Experiment 1).** Error bars represent within-subjects standard errors of the mean.

Two participants were excluded from the sample because they had very high error rates (one had >40% errors on AX trials, the other had 100% errors on AY trials), suggesting that they either failed to understand the instructions or could not perform the task successfully. The final sample included 76 participants. Descriptive statistics for the AX-CPT as a function of task condition are presented in **Table 1** and illustrated in **Figures 1** and **2**.

**Table 1:** Descriptive statistics for the AX-CPT as a function of task condition (Experiment 1).

Dependent variable	Trial type	Baseline condition	Strategy training condition
Average error rate	AX	0.054 (0.055)	0.060 (0.062)
	AY	0.136 (0.103)	0.208 (0.155)
	BX	0.045 (0.066)	0.044 (0.077)
	BY	0.022 (0.042)	0.020 (0.039)
Average RT	AX	404 (65)	381 (63)
	AY	509 (80)	492 (85)
	BX	378 (96)	341 (78)
	BY	373 (72)	349 (86)
PBI-errors		0.376 (0.365)	0.503 (0.356)
PBI-RTs		0.155 (0.074)	0.184 (0.055)
*d′*-context		3.34 (0.67)	3.39 (0.86)
A-cue bias		0.297 (0.288)	0.410 (0.345)

*Average values with standard deviations in parentheses. PBI-errors, PBI calculated on errors; PBI-RT, PBI calculated on median response times.*

Relative to the baseline AX-CPT, participants in the strategy training condition were expected to demonstrate greater proactive control, as indicated by worse AY performance, better BX performance, and higher PBI, *d′*-context, and A-cue bias values. All analyses were conducted using the general linear model. For error rates, the main effect of task condition (baseline or strategy training) was significant, *F*(1,75) = 5.76, *MSE* = 0.009, *p =* 0.019, η_p_^2^ = 0.07, indicating higher error rates after strategy training. Importantly, the two-way interaction between task and trial type was significant, *F*(3,225) = 12.10, *MSE* = 0.004, *p <* 0.001, η_p_^2^ = 0.14, indicating that the pattern of performance differed in the baseline and the strategy training conditions (**Figure 1**). As predicted, follow-up *t*-tests revealed that participants made more errors on AY trials after strategy training, *t*(75) = -3.95, *p* < 0.001, η_p_^2^ = 0.17, indicating an increased tendency to use proactive control in this condition. Surprisingly, however, error rates on BX trials were unaltered, *t*(75) = 0.08, *p* = 0.933, η_p_^2^ = 0.00.

For RTs, the main effect of task condition was significant, *F*(1,75) = 17.02, *MSE* = 5512, *p <* 0.001, η_p_^2^ = 0.18, reflecting a general speeding of RTs after strategy training. The two-way interaction between trial type and task condition was also significant, *F*(3,225) = 2.82, *MSE* = 909, *p =* 0.040, η_p_^2^ = 0.04, indicating that strategy training differentially affected RTs for the different trial types (**Figure 2**). Participants were significantly faster after strategy training on both BX and AY trials (both *p*s < 0.05). However, relative to baseline, participants were 36 ms faster on average on BX trials after strategy training (*SD* = 74 ms), but they were only 17 ms faster on AY trials (*SD* = 65 ms). This greater improvement on BX trials is again consistent with an increased tendency to use proactive control after strategy training.

The effect of task condition was significant for the PBI calculated on RTs, *F*(1,75) = 9.85, *MSE* = 0.003, *p =* 0.002, η_p_^2^ = 0.12, the PBI calculated on errors, *F*(1,75) = 6.50, *MSE* = 0.094, *p =* 0.013, η_p_^2^ = 0.08, and the composite PBI, *F*(1,75) = 12.89, *MSE* = 0.200, *p <* 0.001, η_p_^2^ = 0.15, indicating that participants were significantly more likely to use proactive control in the strategy training condition compared with the baseline condition. Importantly, both the PBI calculated on RTs and the PBI calculated on errors were significantly greater than zero in baseline as well as after strategy training (all *p*s < 0.001), indicating a heavily proactive profile in both conditions. There was no effect of task condition on the *d′*-context, *F*(1,75) = 0.21, *MSE* = 0.395, *p =* 0.645, η_p_^2^ = 0.00, reflecting the fact that strategy training did not influence BX error rates. However, the main effect of task condition was significant for the A-cue bias, *F*(1,75) = 5.38, *MSE* = 0.090, *p =* 0.023, η_p_^2^ = 0.07, indicating that participants had a higher tendency to prepare a target response after an A cue following strategy training, consistent with an increased use of proactive control.

One limitation of the procedure used here is that strategy training was confounded with practice: participants always completed the baseline condition first and the strategy training condition second. This confound may be problematic in certain populations: for example, it has been shown that older adults tend to shift toward proactive control with practice in the AX-CPT (Paxton et al., 2006). To control for this potential problem, a final series of analyses was conducted to confirm that the strategy training impacted performance above and beyond practice. The results are detailed in Appendix A. Overall, the data indicated that the effect of strategy training was not attributable to a practice effect: all measures of interest demonstrated a specific effect of strategy training, and none of them showed a shift toward proactive control throughout task blocks prior to training.

### Discussion

The strategy training manipulation appeared to be successful in influencing AX-CPT performance across a number of metrics. In particular, there was a sharp uptick in AY error rates, as well as an increased A-cue bias. These effects suggest that participants were able to implement the instructed strategy, in that they had an increased bias to prepare a target response following the A-cue. This pattern is consistent with increased utilization of proactive control. Regarding RTs, strategy training improved BX trials more than AY trials, also in line with our predictions. This conclusion was further supported by the consistently significant effects in the PBI, an index of proactive control developed in Braver et al. (2009), which reflects a relative shift in the balance of interference from BX trials (reflecting reliance on reactive control) to AY trials (reflecting reliance on proactive control). As such, these findings replicate the results of prior studies investigating the impact of strategy training in increasing proactive control in the AX-CPT (Paxton et al., 2006; Braver et al., 2009; Edwards et al., 2010). Additionally, they extend this prior work by demonstrating that the strategy manipulation can be successfully employed in healthy young adults. Further analyses indicated that these effects were not attributable to practice, and specifically followed strategy training.

The main limitation of Experiment 1 is the within-subjects design, with participants always completing the baseline condition first and the strategy training second. Although supplemental analyses suggested that the observed effects were actually due to strategy training rather than practice, it would be of interest to confirm the results using a between-subjects design; to our knowledge, this has not been attempted with young adults (but see Paxton et al., 2006, for a study with older adults). It is also worth mentioning that participants completed the experiment either for payment or for course credit, which could conceivably lead to different patterns of performance (Botvinick and Braver, 2015); this issue was avoided in Experiment 2 and Experiment 3. Lastly, a surprising aspect of the present results is that all but one of the anticipated effects appeared. We expected to observe direct improvement of BX performance, and a related increase of the *d′*-context, following strategy training. This beneficial effect of proactive control in reducing interference from the X-probe did not occur, despite the significant effects of training on other measures.

One possibility to explain the lack of improvement in BX performance is that participants were already prone to using proactive control at baseline, thus masking any improvement on BX trials; this idea is consistent with the fact that young healthy adults generally serve as a “proactive control group” when compared to older adults or pathological samples (e.g., Paxton et al., 2008; Braver et al., 2009; Edwards et al., 2010). Specifically, if participants already had a strong tendency to use proactive control at baseline, their performance on BX trials may have been high enough that further improvement would have been difficult to notice. Congruent with this idea, examining the descriptive statistics revealed that participants made on average less than 5% of errors on BX trials, which amounts to less than a single error throughout the task. In fact, half the participants (37 out of 79) made no error at all in the baseline condition. By contrast, participants were much slower and made almost three times as many errors on AY trials. In the following study, we explored the use of a task manipulation that was implemented to reduce the utilization of proactive control, and in so doing, to bring BX performance off ceiling.

## Experiment 2

Experiment 1 suggested that strategy training is an effective method to induce participants to use proactive control. Conversely, the first objective of Experiment 2 was to validate a version of the AX-CPT that decreased the use of proactive control. To this end, additional no-go trials were interspersed throughout the AX-CPT, similar to the procedure used by Braver et al. (2009). In contrast to Braver et al. (2009), the effectiveness of the no-go manipulation was tested in a large sample and without the use of additional monetary incentives. A second objective of Experiment 2 was to determine whether decreasing the utilization of proactive control through no-go trials could take performance off of ceiling levels for BX trials, thus providing a more sensitive assessment of cognitive control.

In no-go trials, the probe was a digit rather than a letter; participants were required to not respond at all whenever they viewed a digit as the probe. Importantly, no-go trials could start with any letter as the cue, which means they were not predictable. As a consequence, the presence of no-go trials reduced the predictive utility of the contextual cues by making response alternatives more uncertain: in contrast to the classic AX-CPT, a trial starting with a B-cue did not automatically require a non-target response, and a trial starting with an A-cue was less likely to require a target response. The introduction of no-go trials was therefore expected to reduce the tendency of participants to utilize proactive control to actively prepare a response during the inter-stimulus interval, and to elicit a corresponding pattern of increased BX interference and decreased AY interference.

### Method

#### Statistical Power

The required sample size was estimated with a power analysis using G^∗^Power 3.1 (Faul et al., 2007). Based on data reported in Braver et al. (2009), we expected an effect size of η_p_^2^ = 0.70 for the effect of no-go trials on the PBI computed on RTs and η_p_^2^ = 0.16 for the composite PBI. The necessary sample size to detect an effect size of η_p_^2^ = 0.16 with 0.95 power was estimated to be *N* = 72. All participants who signed up for the study within 1 week completed the protocol.

#### Participants

A sample of 95 undergraduate students at the University of Savoy completed the experiment in exchange for partial course credit. All participants were native French speakers between the ages of 17 and 25 (*M* = 20.18 years; 21 males and 74 females) with no history of neurological disorders and without psychoactive medication. The approval of an ethics committee was not required for this experiment under local regulations; all participants provided written informed consent prior to completing the protocol, in accordance with the declaration of Helsinki.

#### Materials

##### Baseline AX-CPT

The task was similar to the version used in Experiment 1, with a number of minor task parameter changes. Each trial comprised a cue presented for 500 ms, a 3500 ms delay period, a probe presented for 500 ms, and a 1000 ms unfilled inter-trial interval. Participants were required to respond to all stimuli, including both cues and probes to ensure encoding of the cues (Paxton et al., 2008). Responses were made by pressing either a blue key with the index finger (for cues and for non-target responses) or a yellow key with the middle finger (for target responses). To help participants keep track of the order of stimuli within a trial, cues were selected among a first set of letters (E, G, P, R, S, and A) and probes were selected among a different set of letters (F, J, M, Q, U, and X); cues were always presented in blue font whereas probes were presented in white (for a similar procedure, Henderson et al., 2012). Participants received audio feedback after each response (with three different sounds indicating a correct response, an incorrect response, or a response that was too slow). The relative frequencies of the different trial types was based on Richmond et al. (2015) as in Experiment 1, but with a reduced number of total trials (40 AX, 10 AY, 10 BX, 40 BY).

##### No-Go AX-CPT

The no-go condition of the AX-CPT was identical to the baseline version except that additional no-go trials were interspersed throughout the task. In the no-go trials, the probe took the form of a digit (any digit from 1 to 9) rather than a letter; participants were instructed to withhold their response altogether whenever they observed a letter followed by a digit. The audio feedback included a fourth sound in this condition, indicating an error corresponding to any response on a no-go trial. The go trial types were matched in number and proportion to the baseline condition (40 AX, 10 AY, 10 BX, 40 BY), and an additional 24 no-go trials were added, intermixed with go trials. Half of the no-go trials began with an A cue, and half of the no-go trials began with a B-cue (signaled by any letter other than A). Thus, no-go trials could not be predicted from the cue.

#### Procedure

Participants completed the experimental session in groups of up to eight participants. They completed one block of trials for the baseline version of the AX-CPT and one block of trials for the no-go version; the order of the two blocks was counterbalanced. Participants completed 100 trials in the baseline AX-CPT and 124 trials in the no-go AX-CPT. Trials were presented in the same pseudo-random order for all participants. The order was defined so that there were no series of more than five consecutive AX trials or two consecutive trials of another type. Each task block was preceded by a practice session including 12 trials, with trial frequencies similar to the following task block. The practice session was repeated until participants reached 70% accuracy.

### Results

**FIGURE 3:**
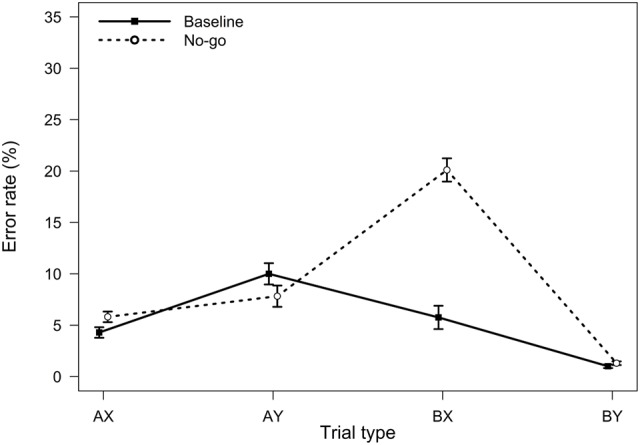
**Average error rates in the AX-CPT as a function of trial type and task condition (Experiment 2).** Error bars represent within-subjects standard errors of the mean.

**FIGURE 4:**
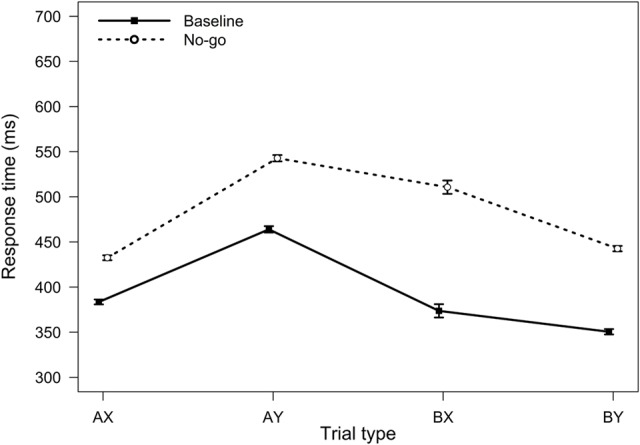
**Average response times in the AX-CPT as a function of trial type and task condition (Experiment 2).** Error bars represent within-subjects standard errors of the mean.

Three participants were excluded from the sample because they had very high error rates (two participants had > 40% errors on AX trials, the third had 100% errors on BX trials). The final sample included 92 participants. Descriptive statistics for the AX-CPT as a function of task condition are presented in **Table 2** and illustrated in **Figures 3** and **4**.

**Table 2:** Descriptive statistics for the AX-CPT as a function of task condition (Experiment 2).

Dependent variable	Trial type	Standard condition	No-go condition
Average error rate	AX	0.043 (0.049)	0.058 (0.062)
	AY	0.100 (0.118)	0.078 (0.091)
	BX	0.058 (0.083)	0.201 (0.158)
	BY	0.010 (0.018)	0.013 (0.021)
	NGA	–	0.164 (0.141)
	NGB	–	0.265 (0.175)
Average RT	AX	384 (43)	433 (55)
	AY	464 (54)	543 (58)
	BX	374 (88)	511 (107)
	BY	351 (59)	443 (52)
PBI-errors		0.113 (0.446)	-0.279 (0.422)
PBI-RTs		0.117 (0.088)	0.038 (0.089)
*d′*-context		3.13 (0.58)	2.46 (0.70)
A-cue bias		0.257 (0.276)	0.163 (0.297)

*Average values with standard deviations in parentheses. NGA, no-go trials starting with an A-cue; NGB, no-go trials starting with any other cue. Average response times were not calculated for no-go trials because making any response on these trials reflected an error. Additionally, errors in no-go trials were not frequent enough to provide a reliable estimate of response times.*

Relative to the baseline condition, participants in the no-go condition were expected to demonstrate reduced proactive control, as indicated by better AY performance, worse BX performance, and lower PBI, *d′*-context, and A-cue bias values. All analyses were conducted using the general linear model. To control for possible order effects, the order of the two task blocks (baseline followed by no-go versus no-go followed by baseline) was entered as a predictor in all analyses; the analyses yielded comparable results without this covariate. For error rates, the main effect of experimental condition was significant, *F*(1,90) = 31.49, *MSE* = 0.007, *p <* 0.001, η_p_^2^ = 0.26, reflecting a higher error rate in the no-go condition. Importantly, the two-way interaction between task block and trial type was also significant, *F*(3,270) = 48.79, *MSE* = 0.006, *p <* 0.001, η_p_^2^ = 0.35, indicating that the pattern of performance as a function of trial type differed in the standard and in the no-go condition of the AX-CPT (**Figure 3**). As predicted, participants made more errors on BX trials in the no-go condition, *F*(1,90) = 86.53, *MSE* = 0.011, *p* < 0.001, η_p_^2^ = 0.49, and marginally fewer errors on AY trials, *F*(1,90) = 3.19, *MSE* = 0.009, *p* = 0.077, η_p_^2^ = 0.03, consistent with a reduced tendency to use proactive control in this condition.

For RTs, the main effect of the task condition was significant, *F*(1,90) = 480.81, *MSE* = 3063, *p <* 0.001, η_p_^2^ = 0.84, reflecting a general slowing in RTs in the no-go condition. The two-way interaction between task block and trial type was also significant, *F*(3,270) = 50.34, *MSE* = 1360, *p <* 0.001, η_p_^2^ = 0.36 (**Figure 4**). The no-go condition elicited slowing on both BX and AY trials (both *p*s < 0.001). However, relative to baseline, participants were on average 137 ms slower on BX trials in the no-go condition (*SD* = 100 ms), whereas they were only 79 ms slower on AY trials (*SD* = 48 ms). These results are again consistent with a reduced tendency to use proactive control.

The PBIs were lower in the no-go condition than in the baseline condition, indicating a lower tendency to use proactive control in the no-go condition; this was true for the PBI calculated on RTs, *F*(1,90) = 64.01, *MSE* = 0.005, *p <* 0.001, η_p_^2^ = 0.42, the PBI calculated on error rates, *F*(1,90) = 57.11, *MSE* = 0.136, *p <* 0.001, η_p_^2^ = 0.39, and the composite PBI, *F*(1,90) = 122.16, *MSE* = 0.279, *p <* 0.001, η_p_^2^ = 0.58. The PBI computed on errors was significantly greater than zero in the baseline (*p* = 0.017) and became significantly lower than zero in the no-go condition (*p* < 0.001), suggesting a decreased reliance on proactive control (or an increased reliance on reactive control). The *d′*-context was also lower in the no-go condition than in the standard condition, *F*(1,90) = 67.56, *MSE* = 0.300, *p <* 0.001, η_p_^2^ = 0.43, reflecting decreased efficiency in using contextual information from the cue to drive responses to the probe in the no-go condition. Lastly, the A-cue bias was lower in the no-go condition than in the baseline condition, *F*(1,90) = 5.89, *MSE* = 0.070, *p =* 0.017, η_p_^2^ = 0.06, indicating that participants were less likely to prepare a target response following an A cue in the no-go condition.

### Discussion

Adding no-go trials to the AX-CPT successfully shifted participants away from using proactive control. As predicted, no-go trials elicited the opposite effect of the strategy training method used in Experiment 1: participants demonstrated worse performance on BX trials and marginally improved performance on AY trials, although the predicted improvement again failed to reach significance. The no-go manipulation also slowed BX trials more than AY trials, consistent with our predictions. Importantly, the fact that the manipulation differentially affected AY and BX trials confirms that it had an effect on proactive control: if no-go trials had merely increased task demands by introducing a new trial type, we would have expected to observe lower performance on all trial types indiscriminately. Derived measures such as the PBI also confirmed reduced utilization of proactive control in the presence of no-go trials, thereby replicating prior work by Braver et al. (2009). The current results extended the prior work by showing the validity of the manipulation in a much larger sample, and by removing monetary penalties for no-go errors. Thus, the success of the no-go manipulation is not dependent on the presence of motivational incentives or punishment. These results were also replicated in an additional experiment conducted in parallel with Experiment 2 (see Appendix B).

Importantly, the results also demonstrated that the no-go manipulation was successful in taking BX performance off of ceiling levels, as reflected by the reduction in *d′*-context and increase in BX interference. This finding suggests that the no-go AX-CPT might be a more sensitive paradigm than the traditional version to clearly detect the influence of task manipulations such as proactive strategy training in healthy young adults. In Experiment 3, we took advantage of this possibility to provide a more powerful test of the effects of strategy training on proactive control.

## Experiment 3

Experiment 1 suggested that strategy training could be used to induce participants to utilize proactive control, whereas Experiments 2 indicated that including no-go trials in the AX-CPT reduced the reliance on proactive control. The objective of Experiment 3 was to strengthen our conclusions by combining these two approaches. Specifically, we hypothesized that in Experiment 1 participants may have already been fairly successful at engaging proactive control under baseline conditions, which would make the strategy training manipulation less sensitive in detecting changes in these processes. If this interpretation is correct, it should be possible to observe stronger effects of strategy training on proactive control if participants were not at ceiling levels at baseline. Based on the results of Experiment 2, one potentially powerful approach would be to treat the no-go condition as the AX-CPT baseline, and apply strategy training to this condition. With this condition as the baseline, we predicted a more substantial effect of proactive control training, in terms of not only increased AY interference, but also improved performance on BX trials. Experiment 3 tested this hypothesis. Participants completed a baseline AX-CPT including no-go trials, underwent strategy training, and completed the same version of the AX-CPT a second time. Thus, Experiment 3 provided an integrative test of the effect of no-go trials and strategy training. As a secondary means of establishing the validity of the strategy training, participants also completed a short questionnaire evaluating their perception of the training and its influence on their behavior during the task.

### Method

#### Statistical Power

The required sample size for this study was estimated with a power analysis using G^∗^Power 3.1 (Faul et al., 2007). Effect sizes for the comparison between no-go and baseline conditions in Experiment 2 were on average η_p_^2^ = 0.35; a comparison between no-go and strategy training conditions was expected to yield even stronger effects. The necessary sample size to detect an effect size of η_p_^2^ = 0.35 with 0.95 power was estimated to be *N* = 28. Funding was allocated for 35 participants, who all completed the study.

#### Participants

A sample of 35 students at Washington University in Saint Louis completed the experiment in exchange for payment ($10.00 an hour). All participants were native English speakers (12 males and 23 females; mean age = 20.90 years). The experiment was approved by an ethics committee (Washington University institutional review board); all participants provided written informed consent in accordance with the declaration of Helsinki.

#### Materials

##### AX-CPT

The task was similar to the version presented in Experiment 2, with the following changes: the delay period was lengthened to 4500 ms, the inter-trial interval was lengthened to 1500ms, and a larger set of cue and probe letters was used (*A, C, D, E, F, G, H, M, N, P, T, U*, and *X*), excluding all letters bearing a perceptual similarity with digits. No-go trials were present in the task, similar to Experiment 2: these trials were signaled by the probe being a digit (any digit from one to 9nine) rather than a letter. The frequencies of the different trial types within each task block were comparable to Experiment 2.

##### Strategy training

The strategy training was similar to Experiment 1. Participants were first informed that an A cue would be followed by an X probe 80% of the time. They were then asked to mentally prepare the most likely response to the probe during the interval following the cue. Lastly, participants were trained to implement this advance preparation strategy during practice trials. In a first phase, the experimenter completed 14 practice trials by pressing the response buttons; at the same time, the participant had to say out loud “yellow” (indicating the target response key) during the delay period if the cue was an A and “blue” (indicating the non-target response key) if the cue was not an A. In a second phase, the participant completed a series of 24 practice trials by pressing the response buttons themselves; they still had to say “yellow” or “blue” out loud during the delay period.

##### Strategy training questionnaire

To ensure that the strategy training had an actual influence on the strategy used in the task, participants were required to complete a short questionnaire at the end of the session. The questionnaire comprised five questions; participants had to respond to each question by indicating their answer on a 9-point scale ranging from “not at all” to “completely.” The following questions were asked in random order:

(1) Did you try to follow the strategy instructions during the task?
(2) Do you think the strategy training influenced the way you performed the task?
(3) Did you have trouble following the strategy instructions during the task?
(4) Were you already using the advance preparation strategy in the previous version of the task?

#### Procedure

Participants completed the testing session individually. The protocol was divided into two sessions separated by 1 week; participants completed the no-go version of the AX-CPT in the first session and the strategy training version in the second session. They completed four blocks of 48 trials for each version of the AX-CPT, for a total of 192 trials per condition, each composed of 160 go-trials (64 AX, 16 AY, 16 BX, 64 AY) and 32 no-go (16 preceded by A-cue, 16 by B-cue). Trials were presented in random order with the constraint that there could be no more than two consecutive AY trials, BX trials, no-go trials with an A cue, or no-go trials with a B cue. The no-go AX-CPT was preceded by 12 practice trials, which were repeated until the participant reached at least 70% accuracy. Participants took a short break between each task block.

### Results

**FIGURE 5:**
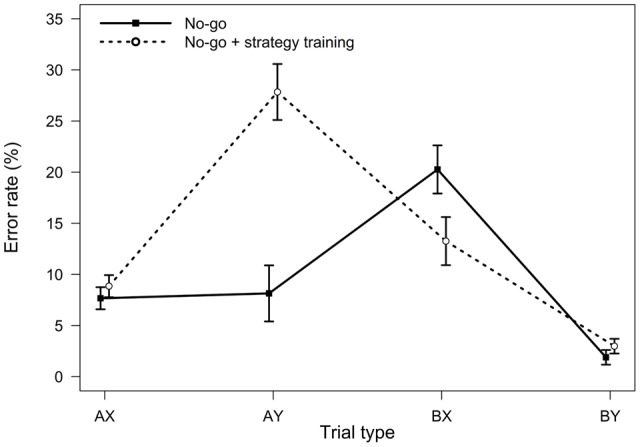
**Average error rates in the AX-CPT as a function of trial type and task condition (Experiment 3).** Error bars represent within-subjects standard errors of the mean.

**FIGURE 6:**
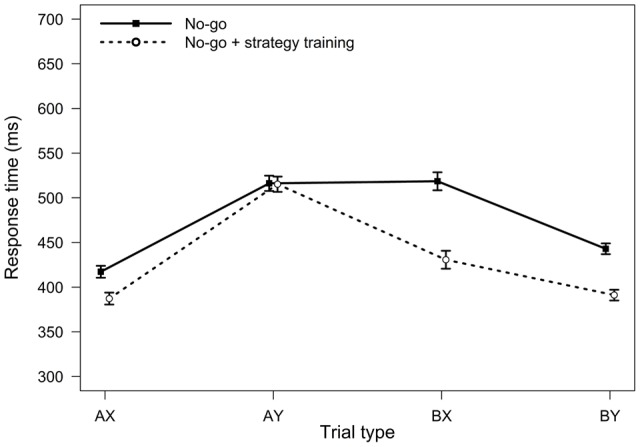
**Average response times in the AX-CPT as a function of trial type and task condition (Experiment 3).** Error bars represent within-subjects standard errors of the mean.

Two participants were excluded from the sample because they had very high error rates (both had >40% errors on AX trials). The final sample included 33 participants. Descriptive statistics for the AX-CPT as a function of task condition are presented in **Table 3** and illustrated in **Figures 5** and **6**.

**Table 3:** Descriptive statistics for the AX-CPT as a function of task condition (Experiment 3).

Dependent variable	Trial type	No-go condition	No-go + strategy training condition
Average error rate	AX	0.077 (0.074)	0.089 (0.106)
	AY	0.081 (0.094)	0.278 (0.245)
	BX	0.203 (0.155)	0.133 (0.139)
	BY	0.019 (0.041)	0.030 (0.060)
	NGA	0.100 (0.100)	0.248 (0.228)
	NGB	0.180 (0.137)	0.373 (0.254)
Average RT	AX	417 (51)	387 (72)
	AY	516 (43)	515 (78)
	BX	519 (102)	431 (99)
	BY	443 (47)	391 (65)
PBI-errors		-0.293 (0.457)	0.219 (0.552)
PBI-RTs		0.005 (0.084)	0.096 (0.076)
*d′*-context		2.44 (0.88)	2.73 (1.04)
A-cue bias		0.083 (0.311)	0.419 (0.480)

*Average values with standard deviations in parentheses. NGA, no-go trials starting with an A-cue; NGB, no-go trials starting with any other cue. Average response times were not calculated for no-go trials because making any response on these trials reflected an error. Additionally, errors in no-go trials were not frequent enough to provide a reliable estimate of response times.*

Relative to the baseline no-go condition, participants were expected to demonstrate increased proactive control in the no-go + strategy training condition, as indicated by worse AY performance, better BX performance, and higher PBI, *d′*-context, and A-cue bias values. That is, we expected to replicate the proactive strategy training results of Experiment 1, with the addition of demonstrating the decrease in BX errors that was not found in Experiment 1.

For error rates, the main effect of the experimental condition was significant, *F*(1,32) = 5.16, *MSE* = 0.018, *p* = 0.030, η_p_^2^ = 0.14, indicating a higher error rate following strategy training. The two-way interaction between task and trial type was significant, *F*(3,96) = 20.60, *MSE* = 0.010, *p <* 0.001, η_p_^2^ = 0.39, indicating that the pattern of performance as a function of trial type differed in two task conditions (**Figure 5**). As predicted, follow-up *t*-tests indicated that participants made more errors on AY trials in the strategy training condition, *t*(32) = -5.08, *p* < 0.001, η_p_^2^ = 0.45, and less errors on BX trials, *t*(32) = 2.11, *p* = 0.043, η_p_^2^ = 0.12, consistent with an increased tendency to use proactive control in this condition.

For RTs, the main effect of task condition was significant, *F*(1,32) = 25.12, *MSE* = 4783, *p <* 0.001, η_p_^2^ = 0.44, indicating faster RTs in the strategy training condition. The two-way interaction between task and trial type was also significant, *F*(3,96) = 18.23, *MSE* = 1213, *p <* 0.001, η_p_^2^ = 0.36 (**Figure 6**). Relative to baseline, participants were significantly faster on BX trials after strategy training (on average 88 ms faster, *SD* = 81 ms, *p* < 0.001), but not on AY trials (on average 1 ms faster, *SD* = 70 ms, *p* = 0.936), consistent with an increased tendency to use proactive control after strategy training.

The effect of task condition was significant for the PBI calculated on RTs, *F*(1,32) = 36.02, *MSE* = 0.004, *p <* 0.001, η_p_^2^ = 0.53, the PBI calculated on errors, *F*(1,32) = 25.37, *MSE* = 0.171, *p <* 0.001, η_p_^2^ = 0.44, and the composite PBI, *F*(1,32) = 61.43, *MSE* = 0.258, *p <* 0.001, η_p_^2^ = 0.66, indicating that participants were significantly more likely to use proactive control following the strategy training. Interestingly, the PBI computed on errors was significantly lower than zero in the no-go condition, *t*(32) = -3.69, *p* < 0.001, η_p_^2^ = 0.30, and became significantly higher than zero after strategy training, *t*(32) = 2.28, *p* = 0.029, η_p_^2^ = 0.14. The PBI computed on RTs did not differ from zero in the no-go condition, *t*(32) = 0.35, *p* = 0.728, η_p_^2^ = 0.00, and also became significantly higher than zero after training, *t*(32) = 7.32, *p* < 0.001, η_p_^2^ = 0.63. As in Experiment 1, there was no effect of task condition on the *d′*-context, *F*(1,32) = 2.10, *MSE* = 0.634, *p =* 0.157, η_p_^2^ = 0.06, but the A-cue bias was significantly higher after strategy training, *F*(1,32) = 16.79, *MSE* = 0.111, *p <* 0.001, η_p_^2^ = 0.34, indicating that participants were more likely to prepare a target response following an A cue after training.

The post-task questionnaire largely confirmed the behavioral performance results. On average, participants reported that they tried to follow the strategy instructions (*M* = 5.85, *SD* = 1.35, range = 1–7 out of a possible 9) and did not have particular trouble doing so (*M* = 2.64, *SD* = 1.56, range = 1–6 out of 9, where higher numbers indicate more difficulty following the instructions). Participants also reported that they were already preparing their responses in advance in the no-go AX-CPT a moderate amount (*M* = 5.48, *SD* = 1.66, range = 1–9 out of 9), but that the strategy instructions also moderately influenced the way they performed the task (*M* = 5.00, *SD* = 1.60, range = 1–8 out of 9).

### Discussion

The results of Experiment 3 not only replicated, but also extended those of Experiments 1 and 2: participants demonstrated a pattern of performance consistent with reduced proactive control in the no-go condition, but strategy training was successful in shifting performance toward utilization of proactive control, as evidenced by the increased A-cue biasing effects that were observed on both AY error rates and the A-cue bias signal detection measure. Importantly, the inclusion of no-go trials in the baseline condition also revealed a significant reduction in BX interference following strategy training; this was reflected not only in a shift of the derived PBI measure, as in Experiment 1, but also in a significantly lower error rate along with selectively faster RTs on BX trials. All effect sizes elicited by strategy training were also much larger than in Experiment 1: for example, the η_p_^2^ for the effect of strategy training on the PBI composite was 0.11 in Experiment 1 and 0.66 in Experiment 3.

In short, these results support the idea that the inclusion of no-go trials can effectively lower the level of proactive control in the AX-CPT, making the task more sensitive to the detection of strategy training effects. As such, the findings indicate that even in healthy young adults, strategy training can provide the full complement of behavioral performance measures previously associated with a shift toward more-proactive control in other populations (i.e., both an increase in A-cue interference and a reduction in X-probe interference), provided that the AX-CPT is made sufficiently sensitive via the inclusion of no-go trials. The post-task questionnaire converged with these findings by indicating that strategy training did influence how participants performed the task and did not pose particular compliance problems.

## General Discussion

In the present study, we validated two different experimental manipulations to bias participants toward or away from proactive control in the AX-CPT. Participants who underwent strategy training demonstrated a shift toward proactive control, thus replicating previous results observed in different populations (Paxton et al., 2006; Braver et al., 2009; Edwards et al., 2010). Conversely, adding no-go trials to the AX-CPT reduced the use of proactive control, also replicating previous results in the literature (Braver et al., 2009).

The results presented here are important in two major ways. Firstly, the effects of the experimental manipulations on the patterns of performance were entirely congruent with the DMC account. In line with the results of Braver et al. (2009), which first suggested that no-go trials could reduce proactive control whereas strategy training could increase proactive control by assessing sustained neural activity, the current results confirm that the DMC framework provides a plausible account of the way cognitive control operates in the context of the AX-CPT. Secondly, our results also establish the validity of the two AX-CPT variants in inducing shifts in the use of proactive control. Both the no-go and the strategy training versions of the AX-CPT had reproducible effects on performance in multiple samples of healthy young adults and, importantly, were observed in a within-subjects design. The two manipulations were relatively simple and elicited large effect sizes for error rates, RTs, and derived measures.

Our results therefore indicate that simple variants of the AX-CPT paradigm can contribute to future investigations of cognitive control mechanisms, by providing experimental situations where the functioning of specific control mechanisms can be selectively observed. The basic principle of altering cognitive control tasks to promote the use of one control mechanism also extends beyond the AX-CPT. For example, it is worth noting that the two experimental manipulations presented here could be generalized to other cognitive control tasks in which participants are required to respond to a target as a function of a cue. One example is the cued task-switching paradigm, where participants typically have to identify one of two probes depending on which instruction is provided in a preceding cue. Participants seem to use proactive control in this paradigm (e.g., Bugg and Braver, 2016), and it would be straightforward to induce proactive control shifts by training participants to actively maintain the cued instruction (Paxton, 2011) or by implementing no-go trials in the task.

An important point of discussion concerns the nature of the relationship of proactive to reactive control. In particular, a critical issue with regard to the DMC framework is whether proactive and reactive control actually reflect partly independent dimensions of control deployment, or instead represent opposite ends of a single dimensional continuum. Most prior studies have implicitly adopted the idea that control shifts are best described as shifts along a unitary continuum (i.e., either toward proactive control or toward reactive control). However, it could also be the case that proactive and reactive control are partly dissociable; that is, a participant could simultaneously use both proactive and reactive control, or only one mechanism, or neither of the two. This idea was described in the original account of the DMC framework (Braver et al., 2007) and preliminary evidence suggests that it might be correct (Gonthier et al., 2016). If it is the case that proactive and reactive control are dissociable, then cognitive control shifts may not be adequately described as shifts from reactive to proactive control or the reverse; instead, it is possible that an experimental manipulation could influence one mechanism but not the other. In other words, no-go trials may decrease the tendency of participants to use proactive control without increasing their reliance on reactive control, whereas strategy training may increase the tendency of participants to use proactive control without affecting their use of reactive control (although participants may tend not to engage in reactive control if they already implement proactive control; see Hutchison et al., 2016). This interpretation does not fundamentally alter the nature of our conclusions – the experimental manipulations presented here do elicit a more-proactive and a less-proactive pattern of results – but it could be interesting for future studies to further explore this line of research.

The idea that proactive and reactive control may be dissociable begs the question of whether it is possible to selectively enhance reactive control, without an associated influence on proactive control. According to the DMC framework, such a manipulation should be possible, and should be characterized in terms of a reduction in X-probe error interference, without the associated increased in A-cue interference, but potentially involving X-probe RT slowing (i.e., a shift from heightened BX errors to relatively slowed BX responding). Indeed, in a prior study, we argued that this behavioral profile best characterized healthy “young-old” adults (65–75 years old; Braver et al., 2005). This possibility also illustrates the critical fact that more attention is needed regarding the precise meaning of the various indices of performance in the AX-CPT. For example, the PBI measure used throughout the present study integrates variation on AY and BX trials into a single index; as a result, this index would conflate variation in proactive and reactive control if the two are in fact dissociable. For this reason, future research on the AX-CPT should strive to achieve better understanding of the precise effects of shifts in proactive and reactive control on performance.

## Conclusion

Through a series of experiments, we demonstrated that it is possible to bias healthy young adults toward or away from proactive control using different variants of the AX-CPT paradigm. The results are congruent with the DMC account, and they suggest that modified versions of the AX-CPT have the potential to contribute to future investigations of the cognitive and neural mechanisms underlying cognitive control.

## Author Contributions

Designed the experiments: CG, BM, AC, and TB. Collected the data: CG, BM, and MC. Analyzed the data: CG, BM, and MC. Wrote the article: CG, BM, MC, AC, and TB.

## Conflict of Interest Statement

The authors declare that the research was conducted in the absence of any commercial or financial relationships that could be construed as a potential conflict of interest.

## Footnotes

1 This repartition of trial types differs from the original AX-CPT, which includes 70% of AX trials and 10% of each of the other trial types. Similar effects would be expected for the original AX-CPT, and both versions seem to usually yield comparable results. However, the modified version used by Richmond et al. (2015) has the desirable effect of equating the overall frequencies of A cues, B cues, X probes, and Y probes, thus removing possible confounds associated with first-order frequency effects. This design also maintains expectancy effects, since it leaves the conditional probability of target responses relatively unaffected (e.g., 80% of A cues are followed by an X in the modified version, as opposed to 87.5% in the original AX-CPT).

2 This formula for response bias actually follows Snodgrass and Corwin (1988) rather than the more usual formula (-½^∗^(Z[H]+Z[F]). This is to emphasize that more liberal target response biases would be associated with higher positive numbers.
