# Supplementary material for: Inducing Proactive Control Shifts in the AX-CPT
Source: Front Psychol. 2016 Nov 22;7:1822. doi: 10.3389/fpsyg.2016.01822 (PMC5118587; doi:10.3389/fpsyg.2016.01822)
Supplement: Supplementary file 1 [file Data_Sheet_1.docx]

**Appendix A: Supplemental Information for the Experiments**

**Overview of Procedural Changes across Experiments**

The major features of each experiment are summarized in Table A1. Other minor parameters were also changed across experiments (e.g., the inter-trial interval was 1000ms in Experiment 1 and Experiment 2 and 1500ms in Experiment 3; cues and probes were sampled from a set of 23 letters in Experiment 1, 12 letters in Experiment 2 and 13 letters in Experiment 3; participants completed 10 practice trials in Experiment 1 and 12 practice trials in Experiments 2 and 3). Importantly, all features of the tasks used in the present study correspond to variants of the AX-CPT that have been used prior studies. There is no a priori reason to suspect that these procedural changes had a strong influence on the results. For example, the duration of the delay between cue and probe varied between 3500ms and 4500ms in the present experiments, but such durations remain relatively typical for the AX-CPT literature: past studies have used extreme delay periods ranging from 1000ms to 7500ms or more (e.g. Braver et al., 2005; Paxton et al., 2008; Redick & Engle, 2011).

Table A1

*Main procedural changes across the three experiments*

| Experiment | Number of trials | Presentation times | Delay between cue and probe | Response to the cue required? | Feedback present? |
| --- | --- | --- | --- | --- | --- |
| Experiment 1 | 200 (BA) 150 (ST) | 1000ms cue 500ms probe | 4000ms | No | No |
| Experiment 2 | 100 (BA) 124 (NG) | 500ms cue  500ms probe | 3500ms | Yes | Yes |
| Experiment 3 | 192 (NG) 192 (NG+ST) | 500ms cue  500ms probe | 4500ms | Yes | Yes |

*Note.* BA = baseline condition; ST = strategy training condition; NG = no-go condition.

**Experiment 1 Method**

**Strategy training procedure.** At the beginning of the strategy training, a question first appeared on the screen asking participants to guess the percentage of time that an X appeared as the second letter after seeing an A as the first letter. After typing in their response, the next screen revealed that when the first letter was an A, an X would appear following 80% of the time. Participants were told that the experimenters were interested in whether receiving this information and completing a related strategy training would improve performance.

Each participant was then partnered with another present participant (in the case of an odd number of participants, the experimenter served as the partner). The experimenter fanned out differently colored cards with strategies and asked each pair to choose a card and follow the instructions. This aspect of the procedure was designed simply to encourage strategy deployment; all participants completed the same strategy training. Participants were instructed to mentally prepare their response to the probe during the inter-stimulus interval. Mental preparation was described as thinking “target button” if they saw an A as the first letter and being ready to press the target button, and thinking “non-target button” if they saw a letter other than A as the first letter and being ready to press the non-target button. Participants were told that they might make mistakes on some trials, and while they should try not to, we were primarily interested in whether they could successfully follow the strategy, even if it caused some additional errors.

Lastly, each pair of participants chose an “active” partner and a “support” partner. The active partner completed an abbreviated block (30 trials) attempting to mentally prepare for the probe. The support partner sat next to the active partner during the abbreviated block and physically represented the mental strategy. In other words, the support partner watched the active partner’s screen, and when the support partner observed an A as the first letter, they indicated preparing to press the target button by “pressing down” their index finger in the air within the active partner’s field of vision; when the support partner observed a letter other than A as the first letter, they indicated preparing to press the non-target button by “pressing down” their middle finger in the air within the active partner’s field of vision. At the completion of the 30 trials, the two partners switched roles and completed another block of 30 trials on the other partner’s computer. Following the strategy training, all participants were instructed to complete the task independently, while attempting to mentally prepare for the probe in the way they had practiced.

**Experiment 1 Results**

**Analysis of practice effects.** Because the order of task blocks was not counterbalanced, the effect of strategy training was confounded with practice effects in Experiment 1. To confirm that strategy training impacted performance above and beyond practice, the effect of practice was tested by examining the timecourse of performance throughout the eight task blocks completed by participants. The analyses used a piecewise regression model. Performance was modeled as a function of the experimental condition (baseline vs. strategy training) and task block number (treated as a continuous predictor, thus testing for practice-related linear changes throughout the task). Performance was expected to vary as a function of experimental condition, independently of task block number. Analyses were conducted separately for the PBI indices, the *d'*-context and the A-cue bias to investigate the effect of strategy training on the use of proactive control, and for RTs and error rates on BY trials to investigate the effect of practice on raw performance.

Five competing models were tested: a model in which performance was held constant throughout the task (Model 1), a model in which performance varied in a linear fashion throughout task blocks without an effect of experimental condition (Model 2), a model in which performance only varied as a function of experimental condition (Model 3), a model with additive effects of task block and experimental condition (Model 4), and a model with interactive effects of task block and experimental condition (Model 5). To account for dependency in the data, mixed-effects modeling was used: all models included a random effect for participants (in other words, a separate intercept was estimated for each participant). The models were fit using the *lme4* package for the R statistical environment (Bates, Maechler, Bolker, & Walker, 2015). Model parameters were estimated using maximum likelihood, and the relative fit of the models was assessed using the Bayesian Information Criterion (BIC). The results are summarized in Table A2.

Table A2

*Estimated fit (BIC) for competing models assessing the timecourse of performance as a function of task block and experimental condition*

| Model | PBI-comp | PBI-RTs | PBI-errors | A-cue bias | *d'*-context | BY errors | BY RT |
| --- | --- | --- | --- | --- | --- | --- | --- |
| Model 1 (intercept only) | 1308.0 | 1068.0 | 925.4 | 830.9 | **1529.2** | 1900.6 | 6666.3 |
| Model 2 (intercept+block) | 1294.9 | 1068.5 | 919.5 | 830.0 | 1530.8 | 1896.3 | 6657.1 |
| Model 3 (intercept+condition) | **1289.8** | 1072.4 | **918.6** | **829.9** | 1535.0 | 1894.6 | **6656.0** |
| Model 4 (intercept+block+condition) | 1296.0 | 1066.0 | 924.3 | 835.7 | 1533.2 | 1891.2 | 6661.5 |
| Model 5 (intercept+block*condition) | 1302.2 | **1060.0** | 929.5 | 841.2 | 1535.9 | **1887.4** | 6662.0 |

*Note.* Lower values of the BIC indicate better fit. The best-fitting model for each dependent variable is in bold.

Overall, an effect of experimental condition appeared for all measures of interest, independently of practice. Model 3 demonstrated the best fit for the composite PBI, the PBI computed on errors, the A-cue bias, and RTs on BY trials, indicating only an effect of strategy training with no practice effect. The timecourse of the composite PBI throughout the task is represented in Figure A1 for illustration. Model 5 provided the best fit for the PBI computed on RTs and for error rates on BY trials, indicating both an effect of strategy training and an effect of practice; however, the slope for the effect of practice was weakly negative for the PBI and weakly positive for error rates, suggesting that participants actually had a slight tendency to become less proactive and to make more errors as the task progressed. Model 1 provided the best fit for the *d'*-context, confirming that this index did not vary throughout the task, consistent with previous analyses.





*Figure A1*. Timecourse of the composite PBI throughout task blocks (Experiment 1). Solid lines represent the slope for the regression of composite PBI on task block number, in the baseline condition and in the strategy training condition. The vertical dashed line represents the boundary between the two testing sessions.

**References**

Bates, D., Maechler, M., Bolker, B., & Walker, S. (2015). Fitting Linear Mixed-Effects models using lme4. *Journal of Statistical Software*, *67*(1), 1-48. doi:10.18637/jss.v067.i01.

**Appendix B: Supplemental Experiment**

Experiment 2 suggested that no-go trials were successful at reducing the reliance of participants on proactive control. To provide converging evidence for this conclusion, a supplemental experiment was conducted in parallel with Experiment 2. An independent sample of participants completed a version of the no-go AX-CPT similar to Experiment 2; the data collected with this version of the task were contrasted with the data of the baseline condition in Experiment 1. The procedure and the hypotheses were identical to Experiment 2.

**Method**

**Participants.** A sample of 56 students at Princeton University completed the experiment in exchange for partial course credit or payment ($12.00 an hour). All participants were native English speakers between the ages of 18 and 24 (*M* = 19.7 years; 15 males and 41 females). The experiment was approved by an ethics committee (Princeton University institutional review board); all participants provided written informed consent in accordance with the declaration of Helsinki.

**Materials.**

***No-go AX-CPT.*** The no-go AX-CPT version was identical to the baseline version used in Experiment 1, with the exception that additional no-go trials were interspersed throughout the task. In the no-go trials, the probe took the form of a digit (any digit from 2 to 9) rather than a letter. The instructions to participants were similar to those provided in Experiment 1: participants were instructed to press the target button with the middle finger of their right hand as quickly as possible whenever they observed the AX sequence and to press the non-target key with the index finger of their right hand as quickly as possible whenever they observed any other letter pair. During no-go trials, participants were instructed to not respond at all whenever they observed a letter followed by a digit. As in Experiment 2, the go trial types were matched in number and proportion to the baseline condition (80 AX, 20 AY, 20 BX, 80 BY), and an additional 40 no-go trials were added, intermixed with go trials. Half of the no-go trials began with an A cue, and half of the no-go trials began with a B-cue (signaled by any letter other than A).

**Procedure.** Participants completed a single testing session in groups of up to 6 participants where they performed the no-go variant of the AX-CPT. After a demonstration and 10 practice trials, they completed four blocks of 60 trials, for a total of 240 trials.

**Results**

One participant was excluded from the sample because of a very high error rate (> 40%) on AX trials; the final sample included 55 participants. The data were analyzed by comparing the results collected for the present experiment with the data collected using the baseline AX-CPT in Experiment 1 (*n* = 76). Descriptive statistics for the AX-CPT as a function of task condition are presented in Table B1.

Relative to the baseline condition, participants in the no-go condition were expected to demonstrate reduced proactive control, as indicated by better AY performance, worse BX performance, and lower PBI, *d'*-context, and A-cue bias values. All analyses were conducted using the general linear model. For error rates, the main effect of task condition was significant, *F*(1, 129) = 5.77, *MSE* = 0.012, *p =*.018, η²_p_ = .04, indicating higher error rates in the no-go condition. Importantly, the two-way interaction between experimental condition and trial type was also significant, *F*(3, 387) = 14.91, *MSE* = 0.004, *p <*.001, η²_p_ = .10, indicating that the pattern of performance as a function of trial type differed in the baseline and the no-go condition of the AX-CPT (see Figure B1). As predicted, follow-up *t*-tests indicated that participants in the no-go condition made more errors on BX trials, *t*(129) = -4.85, *p* < .001, η²_p_ = .15, consistent with a decreased tendency to use proactive control in this condition. Participants performed descriptively better on AY trials in the no-go condition, but the effect did not reach significance, *t*(129) = 1.44, *p* = .153, η²_p_ = .02.

Table B1

*Descriptive statistics for the AX-CPT as a function of task condition (supplemental experiment)*

| Dependent variable | Trial type | Baseline condition (Experiment 1) | No-go condition |
| --- | --- | --- | --- |
| Average error rate | AX | .054 (.055) | .080 (.069) |
|  | AY | .136 (.103) | .112 (.084) |
|  | BX | .045 (.066) | .121 (.113) |
|  | BY | .022 (.042) | .036 (.040) |
|  | NGA | - | .066 (.074) |
|  | NGB | - | .095 (.097) |
| Average RT | AX | 404 (65) | 497 (106) |
|  | AY | 509 (80) | 645 (107) |
|  | BX | 378 (96) | 637 (159) |
|  | BY | 373 (72) | 558 (119) |
| PBI-errors | | 0.376 (0.365) | 0.008 (.368) |
| PBI-RTs | | 0.155 (0.074) | 0.015 (.072) |
| *d'*-context | | 3.34 (0.67) | 2.34 (0.63) |
| A-cue bias | | 0.297 (0.288) | 0.063 (0.215) |

*Note.* Average values with standard deviations in parentheses. NGA = no-go trials starting with an A-cue; NGB = no-go trials starting with any other cue. Average response times were not calculated for no-go trials because making any response on these trials reflected an error. Additionally, errors in no-go trials were not frequent enough to provide a reliable estimate of response times.

For RTs, the main effect of task condition was significant, *F*(1, 129) = 103.28, *MSE* = 35086, *p <*.001, η²_p_ = .44, reflecting a general slowing in response times in the presence of no-go trials. The two-way interaction between trial type and task condition was also significant, *F*(3, 387) = 89.05, *MSE* = 1818, *p <*.001, η²_p_ = .41, again indicating that the effect of no-go trials varied as a function of trial type (see Figure B2). No-go trials elicited slowing in response times on both BX and AY trials (both *p*s < .001), but this slowing was more pronounced for BX trials (*M* = 259 ms) than for AY trials (*M* = 137 ms), again consistent with a decreased tendency to use proactive control in the no-go condition.





*Figure B1*. Average error rates in the AX-CPT as a function of trial type and task condition (supplemental experiment). Error bars represent standard errors of the mean.





*Figure B2*. Average response times in the AX-CPT as a function of trial type and task condition (supplemental experiment). Error bars represent standard errors of the mean.

The effect of task condition was significant for the PBI calculated on RTs, *F*(1, 129) = 118.03, *MSE* = 0.005, *p <*.001, η²_p_ = .48, for the PBI calculated on errors, *F*(1, 129) = 32.34, *MSE* = 0.134, *p <*.001, η²_p_ = .20, and for the combination of the two, *F*(1, 129) = 99.85, *MSE* = 0.422, *p <*.001, η²_p_ = .44, indicating that participants were significantly less likely to use proactive control in the no-go condition. The effect of task condition was also significant for the *d'*-context, *F*(1, 129) = 74.67, *MSE* = 0.428, *p <*.001, η²_p_ = .37, indicating that participants were less efficient at using cue information to regulate their answer to the probe in the presence of no-go trials. Lastly, the main effect of task condition was significant for the A-cue bias, *F*(1, 129) = 25.95, *MSE* = 0.068, *p <*.001, η²_p_ = .17, indicating that participants had a lower tendency to prepare a target response after an A cue in the no-go condition.

In summary, the results of this supplementary experiment largely replicated the results of Experiment 2. Adding no-go trials to the AX-CPT successfully shifted participants away from using proactive control: derived measures such as the PBIs were all affected in the predicted direction, and participants were more slowed and committed more errors on BX trials than on AY trials. The major difference with Experiment 2 was that the decrease of error rates on AY trials in the presence of no-go trials did not approach significance, an observation that partly led to the design of Experiment 3.
